# Should We Be Concerned about the Association of Diabetes Mellitus and Periodontal Disease in the Risk of Infection by SARS-CoV-2? A Systematic Review and Hypothesis

**DOI:** 10.3390/medicina57050493

**Published:** 2021-05-13

**Authors:** Miguel Angel Casillas Santana, Juan Antonio Arreguín Cano, Alejandro Dib Kanán, Farid Alonso Dipp Velázquez, Paulina del Carmen Sosa Munguía, Gabriel Alejandro Martínez Castañón, Brenda Eréndida Castillo Silva, Carolina Sámano Valencia, Marco Felipe Salas Orozco

**Affiliations:** 1 Maestría en Estomatología con opción terminal en Ortodoncia, Facultad de Estomatología, Benemérita Universidad Autónoma de Puebla, Puebla 72410, Mexico; alejandro.dib@correo.buap.mx (A.D.K.); farid.dipp@correo.buap.mx (F.A.D.V.); brenda.castillosilva@correo.buap.mx (B.E.C.S.); carolina.samano@correo.buap.mx (C.S.V.); 2 Secretaria de Salud del Estado de Guerrero, Chilpancingo, Guerrero 39000, Mexico; 3 Residente de la Maestría en Ciencias Médicas e Investigación, Facultad de Medicina, Benemérita Universidad Autónoma de Puebla, Puebla 72410, Mexico; paulina.sosam@alumno.buap.mx; 4 Facultad de Estomatología, Universidad Autónoma de San Luis Potosí, San Luis Potosí 78290, Mexico; mtzcastanon@fciencias.uaslp.mx

**Keywords:** periodontal disease, type 2 diabetes mellitus, SARS-CoV-2, COVID-19

## Abstract

The objective of this article was to conduct a systematic review of the literature to contrast the existing evidence regarding the relationship between periodontal disease (PD) and diabetes mellitus (DM) with the possibly increased risk of SARS-CoV-2 infection, as well as to establish a hypothesis that explains the ways in which this interaction could take place. A literature search up from 1 January 2020 to 21 March 2021 was conducted in three electronic databases, namely, PubMed, Web of Science, and Scopus, in order to identify studies on periodontal disease alone or in conjunction with diabetes mellitus, reporting any relation with SARS-CoV-2 infection as a primary outcome. Only articles published in the English language were included. Due to the lack of studies, we decided to collect all the theoretical and clinical evidence suggesting a possible biological pathway evidencing the relationship among PD, DM, and SARS-CoV-2 infection. From a total of 29 articles, 12 were included for final review studies (five reviews, two hypotheses, one Special Issue, one perspective, one commentary, one case–control study, and one case report). In addition, this systematic review article hypothesizes the correlation between PD and type 2 diabetes mellitus (T2DM) by expression of angiotensin-converting enzyme 2 (ACE2) in periodontal tissue and the risk of SARS-CoV-2 infection. T2DM is a metabolic disorder characterized by high blood glucose levels resulting from altered insulin secretion or action. Likewise, periodontitis and T2DM are inflammatory disorders with a bidirectional association, and both diseases have a similar immunomodulatory cascade and cytokine profile. ACE2 is a crucial component of the renin–angiotensin system (RAS) and the key factor of entry in the cells by the new SARS-CoV-2. ACE2 is widely distributed in the lung and kidneys, and interestingly has a great distribution in the oral cavity, principally in the tongue and periodontal tissue. ACE2 in periodontal tissue plays a crucial role between health and disease. Moreover, the ACE2/Ang-(1-7)/MasR axis is downregulated in the dysbiotic and inflammatory periodontal environment. Nevertheless, the balance of ACE2 activity is modified in the context of concurrent diabetes, increasing the expression of ACE2 by the uncontrolled glycemia chronic in T2DM. Therefore, the uncontrolled hyperglycemia possibly increases the risk of developing periodontitis and triggering overexpression of ACE2 in periodontal tissue of T2DM patients, with these events potentially being essential to SARS-CoV-2 infection and the development of mild-to-severe form of COVID-19. In this sense, we would like to point out that the need for randomized controlled trials is imperative to support this association.

## 1. Introduction

The new SARS-CoV-2 (severe acute respiratory syndrome coronavirus 2), designated by the International Committee on Taxonomy of Viruses, which causes coronavirus disease 2019 (COVID-19), is a current global health problem affecting all the countries around the world, with infection spreading rapidly due to the high virulence of the virus [1,2]. Globally, as of 1:30 p.m. CEST, 28 March 2021, there have been 126,359,540 confirmed cases of COVID-19, including 2,769,473 deaths, reported by the WHO. As of 25 March 2021, a total of 462,824,374 vaccine doses have been administered [2]. Age, gender, ethnicity, and comorbidities are risk factors that increase the risk of SARS-CoV-2 infection complications and mortality. In this sense, the patients with chronic diseases, principally type 2 diabetes mellitus (T2DM), are the most affected by COVID-19 [3,4]. The different physiological conditions in T2DM patients modify the physiopathology of COVID-19, such as the immune dysfunction associated with impaired glycemic control and an increase in cytokine release associated with an attenuation of interferon response [5] and increased inflammation through SARS-CoV-2-infected monocytes that stimulate production of the pro-inflammatory cytokines IL-1β, IL-6, and TNF [6]; these changes were also recently described in patients treated with angiotensin-converting enzyme (ACE) inhibitors and angiotensin II type I receptor blockers (ARBs) [7]. The expression of angiotensin-converting enzyme 2 (ACE2) is substantially increased in patients with T2DM, and this receptor has been found to be a molecular target used by SARS-CoV-2 to infect human cells [8]. Due to the binding between ACE2 and SARS-CoV-2, there is an increase in Ang II levels, which are associated with viral load. Ang II is involved in the generation of free radicals, the activation of protein kinases, and the recruitment of inflammatory cells, as well as the synthesis and release of cytokines and chemokines, both of which are linked to inflammation, fibrosis, and tissue damage. ACE2 is a RAS system regulator that reduces the inflammatory response by modulating the damaging effects of Ang II and AT1 [9,10,11,12]. Nevertheless, there is still a significant number of infected patients with severe side effects and complications that have no identified risk factors. For example, one of the main comorbidities of T2DM is periodontal disease (PD), and these two conditions have a bidirectional relationship in which the severity of both diseases is increased [13]. Recent reports have demonstrated that PD maintains high levels of ACE2 in experimental models of diabetes [14,15]. Moreover, the imbalance of pathogenic bacteria in the oral cavity is exacerbated by periodontitis. The bacteria in the oral biofilm could be aspirated into the respiratory tract, causing pneumonia or sepsis and increasing the risk of respiratory infections and potentially post-viral bacterial complications. In this sense, respiratory viral infections can predispose patients to bacterial superinfections, leading to increased disease severity and mortality [16,17]. Rather than the virus itself, bacterial superinfections became the major cause of death during the 2009 H1N1 influenza pandemic [18].

The PD immune response facilitates the release of cytokines locally and systemically (cytokine storm) [19,20]. As a result, patients with PD prior to SARS-CoV2 infection are more likely to have elevated cytokine levels, making them more vulnerable to severe and fatal outcomes. Lung tissues from COVID-19 patients release pro-inflammatory cytokines, which are essential in the development of PD [21]. The expression of the same proinflammatory cytokines implicated in hyperglycemia (IL-6, TNF-α, and CRP) has been reported to be associated with oral infection and PD [22].

Inflammation and oxidative stress are also key regulators in the manifestation of diabetes mellitus and PD. Periodontal infection can therefore induce systemic inflammation, which in turn builds up or reinforces chronic insulin resistance. During COVID-19 infection, ACE2 expression is decreased, and this results in an exaggerated activity of Ang II with subsequent insulin resistance, oxidative stress, inflammation, hypertension, and cardiac dysfunction [23].

The aim of this article is to provide a systematic review of the literature to contrast the existing evidence regarding the relationship between PD-DM and the possibly increased risk of SARS-CoV-2 infection as well as presenting a hypothesis on how PD-DM and SARS-CoV-2 are associated.

## 2. Materials and Methods

This systematic review was conducted according to the recommendations of the PRISMA Statement for reporting of systematic reviews and meta-analysis [24]; the PRISMA guideline is considered the standard and all the guidelines were taken into account for the current article. It is important to highlight the complexity of reporting a systematic review combining theoretical and clinical literature where a biological pathway among PD, DM, and SARS-CoV-2 infection is established. Nevertheless, there is a lack of reliable evidence in this field, and clinical guidelines that accurately explain this relationship are urgently needed.

### 2.1. Search Methods for Identification of Studies

We performed a search for studies published from 1 January 2020 to 21 March 2021, and only articles published in the English language indexed in the electronic databases of Pubmed, Web of Science, and Scopus were included. A librarian team with expertise in systematic reviews assisted in the creation of the search strategy. The aim of the search was to find studies focused on identifying the relationship on PD alone or in conjunction with DM reporting any relation with COVID-19. The main key words were a combination of “diabetes mellitus”, “hyperglycemic”, “type 2 diabetes mellitus”, “COVID-19”, “SARS-CoV-2”, “periodontitis”, “periodontal disease”, and “gum disease”.

### 2.2. Selection Criteria

#### 2.2.1. Types of Studies

There is limited information on the PD–DM–COVID-19 relationship due it is still being a recent topic and articles on the subject being scare; therefore, to contrast all the existing theoretical and clinical information, we included 5 review articles, 2 hypotheses, 1 Special Issue, 1 perspective, 1 commentary, 1 case–control study, and 1 case report study. Studies were excluded if they focused exclusively on the diabetes mellitus and COVID-19 relationship.

#### 2.2.2. Data Collection and Analysis

All the articles found using the search strategy were downloaded into a single file, and duplicates were deleted using Rayyan, a web and mobile app for systematic reviews. Four reviewers (M.A.C.S., M.F.S.O., P.C.S.M., and F.A.D.V.) separately screened all the articles by the titles and abstracts, choosing those that were reasonably appropriate for full text reading. Six authors (M.A.C.S., J.A.A.C., B.E.C.S., C.S.V., A.D.K., and G.A.M.C.) independently extracted data from the studies, compared their findings, and resolved disagreements.

#### 2.2.3. Quality Assessment

Due to the absence of clinical articles, we decided to include a variety of theoretical and clinical articles in order to contrast all the existing information in the literature; those narrative review articles were evaluated with SANRA, a scale for the evaluation of quality of narrative review articles [25]. The Joanna Briggs Institute (JBI) Critical Appraisal Checklist was used for text and expert opinion papers [26], the Newcastle-Ottawa Scale (NOS) for case–control studies [27], and the JBI critical appraisal checklist for case reports [28].

## 3. Results

**Figure 1:**
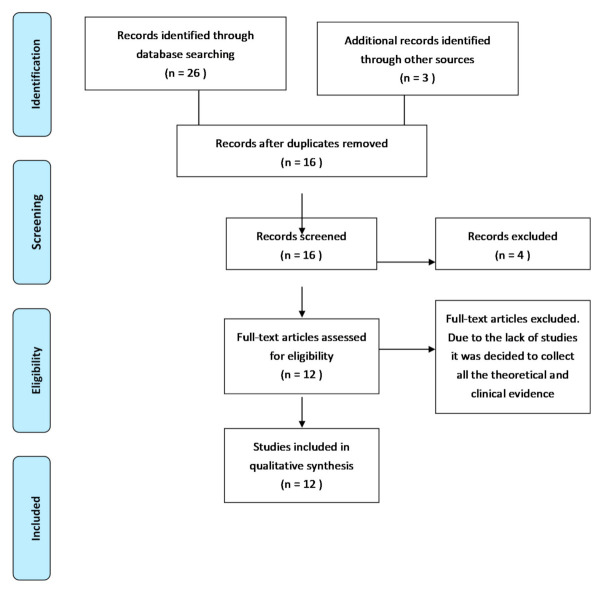
PRISMA flow diagram of record processing and elimination.

The electronic search yielded 29 articles, which were reduced to 16 after the exclusion of duplicates. After title and abstract screening, 4 articles were excluded and 12 were selected for full text reading. A total of 12 studies were included in the review (Figure 1).

### 3.1. Main Findings

Five review articles [29,30,31,32,33], two hypothesis articles [34,35], one perspective article [36], one special issue [37], one commentary [38], one case–control study [39], and one case report [40] were included. Sampson et al. mentioned that the four major comorbidities linked to an increased risk of COVID-19 complications and death are also linked to altered oral biofilms and periodontal disease; oral infections are normal in patients with a serious case of COVID-19, with bacterial superinfections responsible for more than half of all deaths [29]. The proposed mechanisms to explain the potential role of oral bacteria in the pathogenesis of a respiratory infection are as follows: oral pathogens aspiration into the lungs; periodontal disease-associated enzymes can alter mucosal surfaces, allowing respiratory pathogens to bind and colonize; periodontal disease-associated enzymes may destroy the salivary pellicles and hinder bacterial clearance from mucosal surfaces; and periodontal disease-associated cytokines may modify the respiratory epithelium, promoting infection by respiratory pathogens [29]. In the study of Coke et al., the authors described that patients with diabetes are more likely to experience severe symptoms and complications as a result of COVID-19 infection than patients without diabetes. Via ACE2 receptors, hyperglycemia promotes virus entry into cells. Furthermore, PD can be a pre-existing condition that exacerbates COVID-19 outcomes. Inflammation in periodontal infections is often caused by a cytokine storm, which has been linked to adverse outcomes in COVID-19 infections such as acute respiratory disease and multiple organ failure [30]. Moreover, NRF2 (NF-E2-related factor 2) is a redox-sensitive, basic leucine zipper transcriptional factor that upregulates antioxidant gene expression by binding to the promoter region of the antioxidant response element (ARE), also regulating inflammation in the pathogenesis of various disease complications including periodontitis. SARS-CoV-2 inhibits NRF2, indicating that the virus deprives the host cells of an essential cytoprotective pathway [30]. According to Takahashi et al., the presence of periodontopathic bacteria, even when lacking infectivity, are potent proinflammatory stimulants for the lower respiratory tract through aspiration. If patients with mild COVID-19 aspirate periodontopathic bacteria frequently, COVID-19 symptoms may become more severe in combination with viral pneumonia. Moreover, poor oral hygiene can cause COVID-19 aggravation by causing the expression of ACE2, a SARS-CoV-2 receptor, and the expression of inflammatory cytokines in the lower respiratory tract when periodontal bacteria are aspirated. On the other hand, oral care, including periodontal therapy, helps to avoid the onset of pneumonia, as well as the exacerbation of chronic obstructive pulmonary disease [31]. Oral dysbiosis, according to Martu et al., is characterized by the loss of the balance of the oral microbial communities and is related to a variety of oral diseases such as periodontitis, candidiasis, and others. Porphyromonas gingivalis, Tanerella forsythia, Treponema denticola, Prevotella intermedia, Selenomona, and Aggregatibacter are the main bacteria involved in the appearance of periodontitis. Patients with COVID-19 were found to have higher levels of ACE2 on the oral mucosa, as well as an increased presence of Prevotella, Fusobacterium, and Veillonella. In both COVID-19 and periodontitis, the recruitment of cells of inflammation is caused by chemokines. In patients with COVID-19 hospitalized in intensive care units, elevated serum levels of IL-1β, IL-7, IL10, IL-17, IL-2, IL-9, Th17, IFN-γ, GM-CSF, G-CSF, IL-8, TNF-α, MIP1B, MCP1, MIP1A, and IP10 were observed, suggesting that the relation between periodontitis and COVID-19 may be established [32]. According to Campos, the findings on COVID-19 and PD in terms of inflammatory pathways are identical. TNF-α (tumor necrosis factor alpha), IL-1, IL-2, IL-6, and IFN-γ (interferon gamma) were all found to be overexpressed, together with elevated systemic levels of C-reactive protein (CRP), acute phase proteins, coagulation factors, and other aspects related to inflammation. Moreover, the viruses in dental biofilm are distinct in terms of oral health status; dental biofilm viruses in periodontitis tend to be more aggressive [33]. Moreover, pre-exposure of airway epithelial cells to bacteria increases the release of proinflammatory cytokines in response to subsequent viral infection and promotes biofilm development on airway epithelial cells, implying that microbial interactions on pulmonary inflammation have pleiotropic impact. Another issue to consider is periodontitis has been linked to respiratory disorders due to inadequate oral hygiene and a compromised immune system. In addition, oral therapies aimed at controlling oral inflammation and bacterial count have been shown to decrease the prevalence of respiratory illness [33]. Pitones-Rubio et al. stated that PD may be a complication of diabetes caused by an out-of-control level of glycemia, and diabetes raises the risk of developing PD. Moreover, external and internal host factors influence the immune response in both periodontal disease and COVID-19. The proposed mechanisms to understand this association include alterations in vascular, cellular, and host repair processes. In hospitalized patients with serious COVID-19 disease, it was confirmed that intubation harmed their oral health. Other risk factors include the normal or experimental use of medications to combat the SARS CoV-2 virus, a lack of oral hygiene, and other comorbidities that can cause dysbiosis of the oral microbiota, which can lead to oral diseases such as periodontal disease [34]. Obesity also increases the development of reactive oxygen species, which cause oxidative stress, which is significant since oxidative stress is elevated in PD and can lead to its progression. When PD develops in obese people, the spread of bacterial products and proinflammatory cytokines triggers an elevated inflammatory systemic condition [34]. According to Mancini et al., the ACE2–Ang1–7–MasR axis has a key role in the downregulation of the cytokines: IL-6, IL-7, IL-2, TNF-α, IL-1β, and MCP-1, balancing cytokine expression. PD is caused by the same cytokines, but they start in a different manner due to biofilm and bacteria. By binding Ang1–7 and activating MasR, one finds that high levels of ACE2 will promote anti-inflammatory feedback. On the other hand, higher levels of this ACE2, can facilitate the SARS-CoV-2 entry to the oral cavity cells. The virus–ACE2 protein complex triggers a decline in ACE2 levels in infected tissue, which may lead to an increase in cytokine expression in PD [35]. The immune system responds to viral replication by downregulating ACE2 expression, resulting in a variety of acute inflammatory injuries. Reduced ACE2 levels at the cell surface, decreased Ang II degradation, and generation of Ang1–7 may be the product of an ACE2–SARS-COV-2 association. Furthermore, internalization of ACE2 can increase the Ang II/Ang1–7 ratio, potentially exacerbating the inflammatory pattern of the SARS-CoV-2 infection. Dysregulation could exacerbate the progression of inflammatory responses that are subordinated to local overactivity of ACE and Ang II, which promotes dysfunction through COX-2 activation, generating vasoactive prostaglandins and reactive oxygen species (ROS) [35]. The oral cavity, according to Botros et al., is a major source for respiratory infections such as Chlamydia pneumoniae, and patients with periodontal disease are more likely to experience hospital-acquired pneumonia as a complication. Oral pathogens intensify lung infection possibly by aspiration of oral pathogens into the lower respiratory tract [36]. Pfützner et al. mentioned that hyperglycemia destroys the connective tissue in the oral cavity, allowing periodontal fibers to deteriorate. Moreover, the phagocytic function of the mononuclear and polymorphonuclear cells has been shown to be impaired, contributing to the growth of pathogenic subgingival flora; as a result, periodontal infection can trigger systemic inflammation, which can exacerbate insulin resistance. Furthermore, patients who underwent extensive periodontal therapy had a 66% reduced chance of pneumonia on average. When compared to the placebo population, diabetic patients had a 78% higher chance of contracting pneumonia. These results suggest that even in the absence of SARS-CoV-2 infection, the multimorbid patient with diabetes and periodontitis faces a high risk of pneumonia [37]. Kara et al. describes that a similarity between coronavirus (SARS-CoV-2) and galectin-3 (Gal-3) has been discovered. The morphology of an important region in the spike protein of SARS-CoV-2 is almost similar to that of Gal-3, and these spike proteins are necessary for the virus entry into host cells [38]. In a case–control study, Marouf et al. assessed the associations between periodontitis and COVID-19 complications using logistic regression models adjusted for demographic, medical, and behavioral factors. A total of 568 patients were included in the report. Periodontitis was linked to COVID-19 complications such as mortality (OR = 8.81, 95% CI 1.00–77.7), ICU admission (OR = 3.54, 95% CI 1.39–9.05), and need for assisted ventilation (OR = 4.57, 95% CI 1.19–17.4). In COVID-19 patients with periodontitis, blood levels of white blood cells, D-dimer, and C-reactive proteins were all significantly higher [39]. Manzalawi et al. identified three patients from three different Saudi cities who had experienced severe gingival bleeding and pain prior to or concurrent with the confirmation of their COVID-19 infection. Debilitating illness, according to the authors, often leads to a disregard for basic oral hygiene procedures, and COVID-19 is no exception. This causes a spike in dental biofilm accumulation, which is linked to a heightened inflammatory response and clinical symptoms of gingivitis and/or periodontitis. Following the infection’s absence, gingival bleeding decreased significantly [40].

### 3.2. Quality Assessment

The quality assessment results of the articles from Sampson et al., Coke et al., Takahashi et al., Martu et al., and Campos et al. [29,30,31,32,33] are shown in the Table 1. Articles from Pitones-Rubio et al., Mancini et al., Botros et al., Pfützner et al., and Kara [34,35,36,37,38] were evaluated with JBI Critical Appraisal Checklist for text and expert opinion papers, which obtained an overall appraisal of inclusion. We assessed each included study with the following questions: (1) Is the source of the opinion clearly identified? (2) Does the source of opinion have standing in the field of expertise? (3) Are the interests of the relevant population the central focus of the opinion? (4) Is the stated position the result of an analytical process, and is there logic in the opinion expressed? (5) Is there reference to the extant literature? (6) Is any incongruence with the literature/sources logically defended? Table 2 shows the results of the evaluation for the Marouf et al. case–control study [39] according to the Newcastle–Ottawa Scale. An overall appraisal of inclusion was obtained for the case report of Manzalawi et al. [40]; we evaluated the following major components: (1) Were patients’ demographic characteristics clearly described? (2) Was the patient’s history clearly described and presented as a timeline? (3) Was the current clinical condition of the patient on presentation clearly described? (4) Were diagnostic tests or assessment methods and the results clearly described? (5) Was the intervention(s) or treatment procedure(s) clearly described? (6) Was the post-intervention clinical condition clearly described? (7) Were adverse events (harms) or unanticipated events identified and described? (8) Does the case report provide takeaway lessons? The answer options were “yes”, “no”, “uncertain”, and “not applicable”.

### 3.3. Hypothesis

#### 3.3.1. Pathophysiology of COVID-19

**Figure 2:**
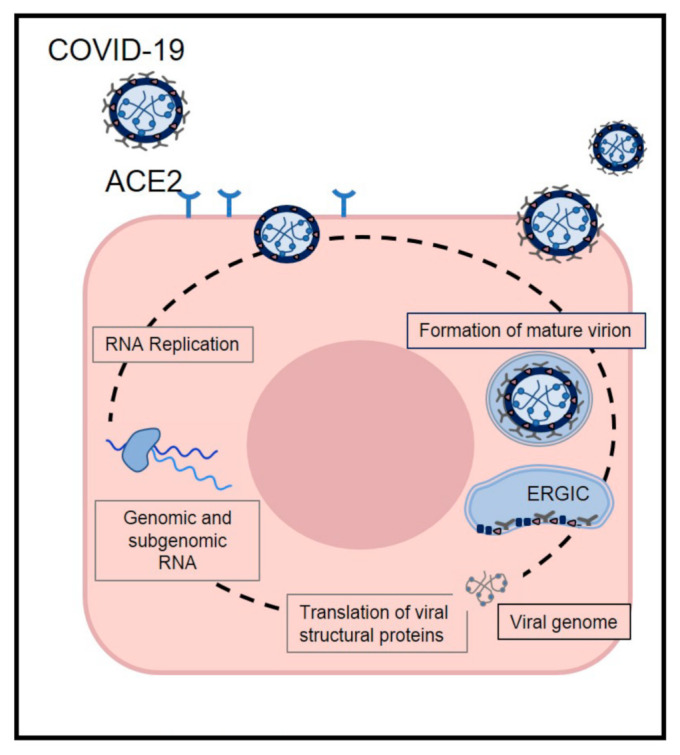
The life cycle of SARS-CoV-2 in host cells. SARS-CoV-2 enters target cells through an endosomal pathway by S proteins binding to cellular receptor angiotensin-converting enzyme 2 (ACE2). Following entry of the virus into the host cells, the viral RNA is unveiled in the cytoplasm. The complex drives the production of negative-sense RNAs through both replication and transcription. Finally viral nucleocapsids are assembled from genomic RNA and N protein in the cytoplasm. Virions are then released from the infected cell through exocytosis.

According to recent studies, 14 days is the common duration for the medical observation and quarantine of exposed persons [41,42]. The common clinical features of COVID-19 include fever, fatigue, dry cough, and myalgia; atypical symptoms, such as headache, anosmia, abdominal pain, diarrhea, and nausea are also observed [43]. The onset of disease may lead to progressive respiratory failure due to alveolar damage and even death [44]. Recent studies indicate that angiotensin-converting enzyme II (ACE2) is likely the target receptor of COVID-19, the same host receptor for SARS-CoV-1 and NL63 [45,46]. The coronavirus S protein can bind to host receptors to facilitate viral entry into target cells, and cellular serine proteases are used for S protein priming. Moreover, it has been demonstrated that overexpressing ACE2 from different cellular lines of HeLa cells allows for COVID-19 infection and replication [47,48]. Nevertheless, COVID-19 does not use other coronavirus receptors but is similar to SARS-CoV-1, which might translate into similar transmissibility and disease pathogenesis [49,50]. Moreover, studies on the biology of viral infection and clinical disease management have demonstrated that differences in COVID-19 prevalence and severity are associated with the high affinity of COVID-19 S protein for ACE2, suggesting that populations with higher expression of ACE2 might be more susceptible to COVID-19 infection [51,52] (Figure 2).

#### 3.3.2. ACE2 Expression in T2DM

Type 2 diabetes mellitus is a metabolic disorder characterized by high blood glucose levels resulting from altered insulin secretion or action [53]. Poor glycemic control increases the inflammatory mediators, such as tumor necrosis factor-α (TNF-α), interleukin-1β (IL-1β), nitrites, and matrix metalloproteinases (MMPs), as well as the expression of the ACE2 receptor [54,55]. These factors evoke a decrease in the immune response of macrophages, increase blood pressure, and reduce the production of collagen by fibroblasts, resulting in delayed tissue recovery and changing the etiopathology of different diseases [56]. These events culminate in acute complications, including ketoacidosis, hyperglycemia, a hyperosmolar state, respiratory infections due to an impaired immune response, periodontal disease, and diabetic coma [57,58]. Therefore, diabetic patients are at increased risk for the development of hypertension, myocardial infarction, renal disease, and stroke [59,60].

The main condition underlying the development of complications in T2DM patients is poor glycemic control, which alters microvascular function in vascular beds in the lungs, kidneys, and periodontal tissue [61,62]. The renin–angiotensin system is implicated in these events and induces vasoconstriction and cellular proliferation [63]. In addition, uncontrolled glycemia decreases surfactant protein A/B levels in the lungs [64], activating the vasoconstrictor component of the renin–angiotensin system, which in turn increases angiotensin II and ACE2 levels in lung tissue [65]. Moreover, in patients with diabetes, the RAS promotes decreased insulin secretion and sensitivity, as well as the progression of diabetic cardiovascular complications [66]. Therefore, high plasma ACE2 activity plays a key role in cardiovascular disease secondary to diabetes.

The expression and distribution of ACE2 in the human body may indicate potential SARS-CoV-2 infection routes, which have major implications for understanding pathogenesis and designing therapeutic strategies [67]. Some studies have shown that ACE2 is potentially expressed in the oral cavity and is highly enriched in epithelial cells [68,69,70,71,72,73,74,75]. According to Xu et al., ACE2 expression was shown to be slightly higher in the oral tongue than in other oral tissues. Moreover, ACE2 is expressed in oral cells (fibroblast and epithelial cells) and immune system cells (B and T cells). The highest levels of ACE2 expression are observed in epithelial cells of the oral tongue [68]. Song et al. reported a moderate expression of the ACE2 gene in the salivary glands. There was no significant difference in ACE2 expression across age groups or between sexes [69]. Via immunohistochemistry, Sakaguchi et al. identified a difference in ACE2 expression between the layers of the tongue’s squamous epithelium. ACE2 has also been found in taste buds’ epithelial cells. The ACE2 gene was also found in the cytoplasm and nucleus of the spinous basal layer of the squamous gingival epithelium. The expression of ACE2 was found in the ductal epithelium and serous cells of the submandibular gland, and the results of RT-PCR showed ACE2 expression in fungiform taste bud cell cultures [70]. Huang et al. found ACE2 expression in different mucosal epithelial cell types: basal 1, basal 2, basal cycling, suprabasal, serous acini, mucous acini, and myoepithelium [71]. The ACE2 gene was shown to be expressed in salivary glands by Pascolo et al., but the ACE2 protein was not found in this oral tissue [72]. The expression of ACE2 in the tongue, lip, and cheek has been stated by Sawa [73]. Chen et al. discovered that ACE2 is expressed in salivary glands [74]. Finally, the immunohistochemistry results reported by Zhong et al. showed that the anatomical areas with the highest expression of ACE2 from highest to lowest were lip, tongue, buccal mucosa, and gingival and palatal tissue. The cells of these anatomical locations that most expressed ACE2 were the epithelial cells of the basal layer, followed by the fibroblasts and the endothelial cells [75]. These findings strongly suggest that the mucosa of the oral cavity may be a potentially high-risk route of SARS-CoV-2 infection.

#### 3.3.3. Do Periodontitis and Type 2 Diabetes Mellitus increase SARS-CoV-2 infection by ACE2?

Periodontitis is a chronic infection-induced inflammatory disease that causes tooth loss and is considered a modifying factor of systemic health [76]. Recent studies have shown a relationship between periodontitis and chronic diseases such as cancer, Alzheimer’s disease, rheumatic arthritis, and T2DM [77,78]. It is unclear if hyperglycemia is merely a marker of disease severity in COVID-19 patients or whether intensive treatment of the hyperglycemia will decrease mortality or other negative outcomes. However, it has been reported that achieving glycemic control soon after admission in both ICU and non-ICU settings can have an effect on outcomes in COVID-19 patients [79]. For the best knowledge of the authors, the currently information mentioned that induced hyperglycemia impairs the mononuclear and polymorphonuclear cells’ phagocytic function, contributing to the growth of violent pathogenic subgingival flora. As a result, periodontal infection can cause systemic inflammation through release of cytokines and metalloproteinases into the circulatory system derived from periodontal disease and tissue destruction, which can exacerbate or worsen chronic insulin resistance [37]. Both diseases have a qualitatively similar immunomodulatory cascade and cytokine profile [80]. Moreover, periodontal disease can increase blood sugar levels, leading to longer periods of time where the body is operating at hyperglycemic conditions, which impairs the function of the innate immune system and may increase viral replication [37]. In this sense, periodontitis is significantly more frequent in individuals with T2DM, and individuals who suffer periodontitis have an increased risk of developing T2DM and diabetic complications [81].

Hyperglycemia can induce nonenzymatic protein glycation, and the resulting advanced glycation end products (AGEs) have been shown to stimulate macrophages, causing them to release cytokines such as IL-6 and TNF-α, as well as C-reactive protein [82]. The expression of these proinflammatory mediators has been linked to oral infection and therefore there is a possibly increased risk of COVID-19 infection in periodontal disease patients. Hyperglycemia also causes individuals more prone to other infections and induces them to deteriorate. Glycemic regulation is thus needed not only in diabetic patients with COVID-19, but also in those who have experienced acute hyperglycemia [82,83]. Moreover, polymorph nuclear leukocytes and monocytes may be induced by periodontal pathogens. This mechanism has the potential to cause oxidative stress and periodontal tissue damage [79].

If the cellular stress is severe, the capacity of the adaptive response to resolve it will be overwhelmed. The integrated stress response (ISR) signaling pathway initiates when distinct stressors activate at least one member of a family of four serine/threonine kinases—PKR-like endoplasmic reticulum kinase (PERK double-stranded RNA-dependent protein kinase (PKR), heme-regulated eIF2a kinase (HRI), and general control non-derepressible 2 (GCN2) [80]. During COVID-19 infection, viral RNA fragments can activate PKR, causing IRS-1 serine phosphorylation and, as a result, insulin resistance. Therefore, it has been suggested that ISR is often accompanied by insulin resistance. Furthermore, the cytokine storm, as well as an improvement in hormonal signaling such as AII and cortisol, will stimulate some of the four kinases, resulting in insulin resistance [84].

Both local and systemic oxidative stress can be exacerbated by periodontal disease. Reactive oxygen species (ROS) synthesis is dramatically enhanced during inflammation, primarily by cells of the innate immune system, such as neutrophils and macrophages [85]. SARS-CoV-2-induced viral pneumonia causes an overactive immune response; almost always, this pathological mechanism is followed by oxidative stress. The viral factors that trigger severe inflammatory responses in macrophages during COVID-19 infection are unclear [86]. Nonetheless, SARS-CoV-2 3a protein displays 72% similarity with SARS-CoV-1 homologue, a non-structured viral protein that activates NLRP3 inflammasome in macrophages, which is accompanied by IL-1β activation and increase in mtROS level. It has been proposed that elevated mtROS production promotes both viral replication and monocyte activation [86].

**Figure 3:**
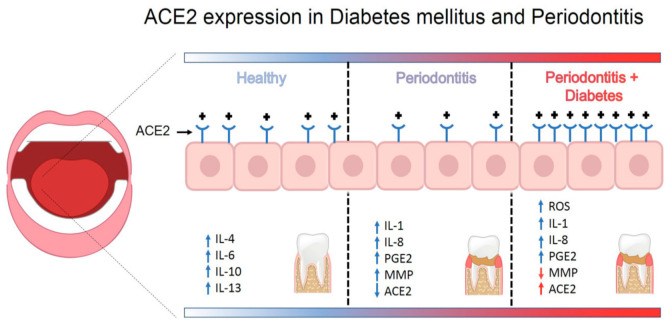
Schematic model for how periodontal disease and type 2 diabetes mellitus augments expression of ACE2 in periodontal tissue.

The RAS system is functional in both healthy and affected periodontal tissue under normal conditions [87]. Likewise, gingival samples from healthy donors show strong expression of ACE2 and MasR [88]. In contrast, ACE2 expression and production are decreased in periodontal disease [89]. Therefore, the ACE2/Ang-(1–7)/MasR axis is downregulated in dysbiotic and inflammatory periodontal environments. Nevertheless, the balance of ACE2 activity is modified in the context of concurrent diabetes. ACE2 and MasR expression and production are higher under conditions of uncontrolled glycemia and periodontitis [90]. These data suggest that ACE2 expression is increased in diabetes associated with periodontitis. On the other hand, the mechanism related to the entry of SARS-CoV-2 into cells is correlated with endocytosis and an internalization of ACE2; consequently, a lower level of ACE2 leads to low degradation of Ang II. The immune system responds to viral replication by downregulating ACE2 expression, resulting in a variety of acute inflammatory injuries. Reduced ACE2 levels at the cell surface, decreased Ang II degradation, and generation of Ang1–7 could be the consequence of an ACE2–SARS-COV-2 association. Furthermore, internalization of ACE2 can increase the Ang II/Ang1–7 ratio, potentially exacerbating the inflammatory pattern of SARS-CoV-2 infection [35]. ACE and Ang II can also promote dysfunction through COX-2 activation, generating vasoactive prostaglandins and reactive oxygen species. Furthermore, Ang II favors the recruitment of infiltrating inflammatory cells into tissues by stimulating the production of specific cytokines/chemokines [35]. Moreover, MMPs have been related to entry and cell-cell fusion of coronavirus [91]. In this sense, a recent study showed the decreases of MMP-2 salivary activity by the uncontrolled glycemia in T2DM patients [55] (Figure 3). Therefore, the uncontrolled hyperglycemia increases the risk of developing periodontitis and triggering overexpression of ACE2, as well as decreased MMP activity in periodontal tissue of T2DM patients; these events could be essential to SARS-CoV-2 infection and development of COVID-19.

Another factor to consider is that people with periodontal disease are more likely to have a complication such as hospital-acquired pneumonia. Oral pathogens can intensify lung infection by aspirating them into the lower respiratory tract [36]. Different respiratory conditions, such as influenza, asthma, and chronic obstructive pulmonary disease, are linked to T2DM. Diabetic patients also have an increased mortality from influenza pneumonia, as seen with the H1N1 virus [83].

Finally, a further aspect that should be investigated is the relationship of ACE2 polymorphisms with diabetes mellitus and periodontitis.

## 4. Strengths and Limitations of this Systematic Review

To the best of the authors’ knowledge, this is the first study of its kind that systematically synthesizes studies that aim to connect PD–DM and the SARS-CoV-2 virus. Moreover, the study establishes a hypothesis that explains the ways in which this interaction could take place. Inclusion of theoretical articles might be considered a potential source of bias in this review; however, collecting and ordering this information could lead to a better understanding of this information. Synthesizing the results of these studies helps provide the necessary information to clinical researchers to conduct randomized clinical trials. Another limitation is that articles were considered only in the English language. Nonetheless, it is believed that although a more extensive search might have identified additional literature, it is unlikely to provide new information in this relationship. Despite the lack of information, this analysis attempted to include a thorough review of the published articles about the PD–DM–SARS-CoV-2 relationship. It is important to highlight that there is good theoretical data linking these three pathologies, and therefore clinical trials to confirm this association are urgently needed.

## Figures and Tables

**Table 1:** Quality assessment results according to SANRA scale.

	Justification of the Article’s Importance for the Readership	Statement of Concrete Aims or Formulation of Questions	Description of the Literature Search	Referencing	Scientific Reasoning	Appropriate Presentation of Data	Sum Score
Sampson 2020	2	2	0	2	2	2	10
Coke 2021	2	2	1	2	2	2	11
Takahashi 2020	2	2	0	2	2	2	10
Martu 2020	1	1	0	1	1	2	6
Campos 2020	2	2	0	2	2	2	10

The six items that form the scale are rated in integers from 0 (low standard) to 2 (high standard), with 1 as an intermediate score. The maximal sum score is 12.

**Table 2:** Newcastle–Ottawa Scale for case–control study results.

J. The Newcastle–Ottawa Scale (NOS) for Case–Control Study	
Major Components	Response Options
Selection	
1. Is the case definition adequate?	
(1) Yes, with independent validation	☆
(2) Yes, e.g., record linkage or based on self reports	
(3) No description	
2. Representativeness of the cases	
(1) Consecutive or obviously representative series of cases	☆
(2) Potential for selection biases or not stated	
3. Selection of controls	
(1) Community controls	☆
(2) Hospital controls	
(3) No description	
4. Definition of controls	
(1) No history of disease (endpoint)	☆
(2) No description of source	
Comparability	
5. Comparability of cases and controls on the basis of the design or analysis	
(1) Study controls for COVID-19 patients	☆
(2) Study controls for any additional factor	☆
Exposure	
6. Ascertainment of exposure	
(1) Secure record (e.g., surgical records)	☆
(2) Structured interview where blind to case/control status	
(3) Interview not blinded to case/control status	
(4) Written self report or medical record only	
(5) No description	
7. Same method of ascertainment for cases and controls	
(1) Yes	☆
(2) No	
8. Non-response rate	
(1) Same rate for both groups	☆
(2) Non-respondents described	
(3) Rate different and no designation	

A study can be awarded a maximum of one star for each numbered item within the Selection and Exposure categories. A maximum of two stars can be given for Comparability.

## Data Availability

Not applicable.
